# Critical appraisal skills training for health care professionals: a randomized controlled trial [ISRCTN46272378]

**DOI:** 10.1186/1472-6920-4-30

**Published:** 2004-12-07

**Authors:** Rod S Taylor, Barnaby C Reeves, Paul E Ewings, Rebecca J Taylor

**Affiliations:** 1 Department of Public Health & Epidemiology, University of Birmingham; Birmingham, UK; 2 Health Services Research Unit, School of Hygiene and Tropical Medicine; London, UK; 3 Somerset Research and Development Support Unit, Taunton & Somerset NHS Trust, Taunton; UK; 4 Health Economics Facility, University of Birmingham, Birmingham, UK

## Abstract

**Introduction:**

Critical appraisal skills are believed to play a central role in an evidence-based approach to health practice. The aim of this study was to evaluate the effectiveness and costs of a critical appraisal skills educational intervention aimed at health care professionals.

**Methods:**

This prospective controlled trial randomized 145 self-selected general practitioners, hospital physicians, professions allied to medicine, and healthcare managers/administrators from the South West of England to a half-day critical appraisal skills training workshop (based on the model of problem-based small group learning) or waiting list control. The following outcomes were assessed at 6-months follow up: knowledge of the principles necessary for appraising evidence; attitudes towards the use of evidence about healthcare; evidence seeking behaviour; perceived confidence in appraising evidence; and ability to critically appraise a systematic review article.

**Results:**

At follow up overall knowledge score [mean difference: 2.6 (95% CI: 0.6 to 4.6)] and ability to appraise the results of a systematic review [mean difference: 1.2 (95% CI: 0.01 to 2.4)] were higher in the critical skills training group compared to control. No statistical significant differences in overall attitude towards evidence, evidence seeking behaviour, perceived confidence, and other areas of critical appraisal skills ability (methodology or generalizability) were observed between groups. Taking into account the workshop provision costs and costs of participants time and expenses of participants, the average cost of providing the critical appraisal workshops was approximately £250 per person.

**Conclusions:**

The findings of this study challenge the policy of funding 'one-off' educational interventions aimed at enhancing the evidence-based practice of health care professionals. Future evaluations of evidence-based practice interventions need to take in account this trial's negative findings and methodological difficulties.

## Introduction

For clinicians to make sense of scientific evidence and follow an evidence-based approach to their practice it has been stated they should be able to: (1) turn problems of their clinical practice into focused questions; (2) comprehensively search for literature to address these questions; (3) critically appraise this literature for its usefulness and scientific validity; and, (4) apply the results of this appraisal to their practice [1].

McColl and colleagues undertook one of the few studies of the prevalence of critical appraisal skills (CAS). In a sample of family practitioners, it was reported that only about one third claimed they "understood and could explain to others" terms which are intimately associated with an ability to critically appraise research [2].

A number of approaches have been developed to help clinicians enhance their CAS, including the publication of a number of critical appraisal checklists and the introduction of CAS teaching into undergraduate and postgraduate education in UK and abroad [3,4]. In UK and abroad, the Critical Appraisal Skills Programme (CASP) has become one of the most widely been disseminated forms of CAS training [5].

Four systematic reviews have been published that explore the effectiveness of CAS training [6-9]. These reviews observed marked heterogeneity in the nature of education intervention across individual studies, particularly in terms of duration (which varied across studies from 1 hour or less to 10 hours or more). However, these reviews consistently reported that CAS training results in small improvements in participants' knowledge of methodological and statistical issues in clinical research and enhances their attitudes towards the use of medical literature in clinical decision making. Nevertheless these findings need to be interpreted with considerable caution as most of the studies had poor internal validity. Only one randomized controlled trial was identified [10] and, in general, studies failed to blind outcome assessment. A focus on classroom-based interventions delivered to either medical students or medical residents, also limits the generalisability of the current evidence base. The aim of this study was to undertake a randomized controlled trial to assess the effectiveness and cost of CAS training in a range of practising healthcare professionals using a range of validated outcomes. Given its wide dissemination, the CASP model of CAS was evaluated in this trial.

## Methods

### Study design

The study was a prospective randomized controlled trial. Study outcomes were not assessed at baseline to avoid a pre-test effect. The possibility of a pre-assessment leading to a higher post assessment score due to an item-practice effect is well recognised in the educational evaluative literature [11]. However, trial participants' characteristics (i.e. gender, age, attitude towards the use of evidence about healthcare research, and details of previous training in research, epidemiology, or statistics) were collected by questionnaire prior to randomization and used as covariates to reduce variation from individual differences. Ethical approval for the study was obtained from all of the local district ethics committees from which the participants were drawn.

### Selection of subjects & setting

Over a three-month period, 1,305 practitioners, working within the South and West Regional Health Authority in England, were sent an invitation to participate in one of a number of CAS workshops being run across the region. Invitations were sent to the health authority offices and all general practices in the geographical area. The letters of invitation included an explanation that agreement to take part in the workshops would include a formal evaluation. Applying to attend, which involved completion of a questionnaire with baseline questions, was taken as consent to enter the study. On receipt of a completed questionnaire, participants were randomized to either intervention or control. The intervention group were given a date to attend a CAS workshop and the control participants assigned to a waiting list to attend a workshop. The only exclusion criterion for entry into the study was attendance at a previous CAS workshop.

### Sample size determination

The target sample size was 200, 100 in each group, which was chosen to allow the study to detect a 'moderate' effect size difference of 0.4 standard deviation units (in any outcome) at 80% power and a 5% significance level (2-tailed) [12].

### Randomization and blinding

An independent researcher used computer generated codes to allocate applicants randomly to intervention (attend a critical appraisal workshop) or control group ('waiting list'), stratified by occupation: manager/administrator; medically qualified practising physician; nurse/profession allied to medicine and 'other' professions. The researchers who scored study outcomes were blinded to the allocation of participants at all times.

### Intervention group

The teaching programme used in this study was based on the Critical Appraisal Skills Programme (CASP). The half-day workshop centres upon facilitating the process by which research evidence is systematically examined to assess study validity, the results and relevance to a particular clinical scenario. Participants practise these skills, during the workshop, by critically appraising a systematic review article and then receive follow up materials following the workshop (see Appendix 1 for details of intervention).

### Development of outcomes

Given the absence of suitable validated outcomes measures, the outcomes were developed for use in trial. A questionnaire was developed and validated (reliability and internal consistency) to assess the following outcomes – knowledge of the principles necessary for appraising evidence; attitudes towards the use of evidence about healthcare; evidence seeking behaviour; perceived confidence in appraising evidence; and, knowledge of the principles necessary for appraising evidence; attitudes towards the use of evidence about healthcare; evidence seeking behaviour; perceived confidence in appraising evidence. A copy of the outcome questionnaire can be found in Appendix 2 (see Additional file 1). Full details of the validation process can be found elsewhere [13].

The questionnaire included 18 multiple-choice knowledge questions, 7 attitude statements and 6 confidence statements. Possible response categories to the knowledge questions were 'true', 'false' or 'don't know'. Correct, incorrect and don't know responses were awarded scores of 1, -1 and 0 respectively. Knowledge scores across question were summed giving a possible range of scores from -18 to +18. Attitude statements were scored on a five-point Likert scale. A 'strongly agree' to a positive attitude statement or 'strongly disagree' to a negative attitude statement was given a score of 5. Conversely, a 'strongly disagree' with a positive attitude statement and 'strongly agree' with a negative attitude statement was give a score of 1. Attitude scores were summed giving a possible range of scores from 7 to 35. The 6 statements of confidence in critical appraisal skills statements were scored using a 1 to 5 Likert scale and summed. A minimum overall score of 5 indicated 'little or no confidence' while a maximum total score of 30 indicated 'complete confidence'.

Critical appraisal ability was assessed through the appraisal of a systematic review article. Participants' critiques were independently assessed by two of the authors (BR & PE) using a 5-point visual analogue scale, a high score indicating a superior level of appraisal skill. A framework for scoring the reviews was developed and agreement assessed; a random sample of 20 appraisals (10 control and 10 intervention) was assessed using this framework. Intra-class correlation coefficients were calculated for each of the three aspects of critical appraisal skills assessed: 'methodology' (0.86), 'results' (0.84) and 'relevance/generalisability' (0.70), indicating satisfactory inter-assessor agreement.

### Assessment of outcomes

Six months after the CAS workshop, the intervention group were asked to complete the outcome questionnaire and undertake the critique of a systematic review article (different to article used in the workshop). Five to six months after randomisation, and about one month prior to attending the workshop, controls were asked to complete the same outcomes. Thus, outcomes were obtained from both groups at about the same time after randomisation.

### Statistical analysis

**Figure 1:**
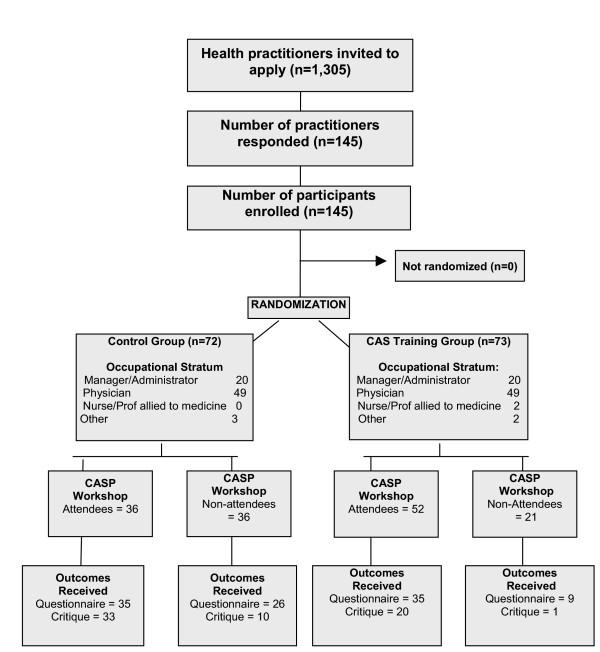
Flow diagram summarising participant recruitment and receipt of outcomes

Primary analysis of the difference between CAS training and control groups was performed on an intention-to-treat basis, adjusting for baseline characteristics. Given that not all participants in the intervention group attended a CASP workshop, a secondary explanatory analysis was also conducted, i.e. according to whether participants received the intervention or not (see Figure 1). For continuous outcomes, multiple linear regression modeling was used to adjust for potential confounding arising from baseline differences in prognostic variables between groups. Regression model goodness of fit was checked by examining model residuals. Ordinal outcomes were compared by Mann-Whitney U tests, and binary outcomes were compared by Chi-squared analyses. Percentages and time variables were analysed as continuous variables. All analyses were carried out using STATA. All statistical tests used a level of significance of 0.05 and two-sided hypothesis testing. 95% confidence intervals (95% CI's) were calculated for differences between the two groups. No adjustment for multiple comparisons was made. However, all analyses were planned *a priori *and reported in full. Costs were analysed using recognized methods [14].

### Cost analysis

A detailed analysis of the costs of setting up and delivering the program of CAS workshops was undertaken. This cost analysis was carried out from the perspective of the NHS. Based on information about the resources and associated costs of providing the workshops, the following items were considered – costs of inviting and processing applications to attend a workshop, time of workshop organizers in the Regional R&D Office, hire of workshop venue and catering, time and expenses of workshop tutors associated with preparing and delivering the workshops, time and expenses (including locum cover) of workshop participants associated with attending the workshops. Published health and social care costs [15], local costs (e.g. NHS trust costs) and Whitley Council pay scale were used to estimate the value of staff time.

## Results

### Subject enrolment

Despite intensive efforts, the trial failed to recruit the target number of individuals. A revised power calculation estimated that, at 5% significance and 80% power, the 145 participants actually recruited would enable the trial to detect a difference of 0.47 standard deviation units (~20% larger than the originally powered difference). 72 were randomized to the control group and 73 to the intervention group. A total of 61 (85%) and 44 (60%) questionnaires and 43 (60%) and 21 (29%) appraisals were returned by the control and CAS training participants respectively (see Figure 1).

The two groups were well balanced for baseline demographic characteristics (see Table 1).

**Table 1:** Distribution of baseline characteristics of health care practitioners randomized to two groups. Values are numbers (percentages) unless otherwise stated.

**Characteristics**	**CAS training N = 73**	**Control N = 72**
**Sex, male**	48 (65.8)	46 (63.9)
**Age (years)**		
<30	2 (2.7)	4 (5.5)
30–39	20 (27.4)	20 (27.8)
40–49	37 (50.6)	32 (44.4)
50–59	12 (16.4)	13 (18.0)
60 +	2 (2.7)	3 (4.2)
**Access to medical library**	71 (97.3)	68 (97.1)
**Prior experience of searching literature**	47 (64.4)	45 (64.3)
Received formal education* in research methods	31 (42.5)	33 (47.1)
Received formal education* in epidemiology	24 (32.9)	22 (31.4)
Received formal education* in statistics	36 (49.3)	39 (55.7)
**Prior involvement in research**	50 (68.5)	41 (58.6)

*: postgraduate education

### Study outcomes

#### 1. Knowledge of the principles necessary for appraising evidence

Participants were asked to answer six knowledge questions, each of which had three parts. The frequency of correct answers to 4 of the 6 questions was higher in the CAS training group than the control. Total knowledge score was significantly higher for the CAS training group than controls [ITT mean difference: 2.6 (95% CI: 0.6 to 4.6); explanatory analysis mean difference 3.1 (95% CI: 1.1 to 5.2)] (see Table 2). A difference in total knowledge score of 2.0 and 3.0 corresponds to difference of 0.2 to 0.3 standard deviation units respectively i.e. below the cut off of 0.4 standard deviations units corresponding to a 'moderate' effect size [12].

**Table 2:** CAS training and control groups total score for knowledge of the principles necessary for appraising evidence, attitude towards the use of evidence, perceived confidence and appraisal skill.

	**CAS training Mean (SD)**	**Control Mean (SD)**	**Intention to treat analysis Mean difference+ (95% CI)**	**Explanatory analysis Mean difference+ (95% CI)**
**Knowledge **[range -18 to 18]	9.7 (5.3)	8.0 (5.1)	2.6 (0.6 to 4.6)*	3.2 (1.1 to 5.2)*
**Attitude **[range 7 to 35]	25.0 (3.8)	24.8 (4.0)	0.04 (-1.5 to 1.6)	-0.04 (-1.7 to 1.6)
**Confidence **[range 6 to 30]	15.0 (5.3)	13.8 (5.1)	1.4 (-0.5 to 3.3)	1.13 (-0.8 to 3.1)
**Appraisal skill **[all range 1 to 5]				
***Methodology***	2.4 (2.5)	2.0 (2.1)	0.6 (-0.8 to 1.9)	0.6 (-0.9 to 2.1)
***Results***	2.6 (2.8)	1.7 (1.8)	1.2 (0.01 to 2.4)*	1.1 (-0.2 to 2.4)
***Relevance/Generalisability***	2.7 (2.2)	2.4 (1.7)	0.3 (-0.8 to 1.4)	0.6 (-0.6 to 1.8)

+ Adjusted for sex, age, attendance at previous educational activity, access to medical library, prior experience of searching literature, formal education in research methods and/or epidemiology and or statistics, prior involvement in research

* Statistically significant at *P *≤ 0.05

#### 2. Attitudes towards the use of evidence about healthcare

With the exception of a more positive response to one attitude statement ('*systematic reviews play a key role in informing evidence-based decisions*'), in the CAS training group compared to control there were no other significant differences between groups in attitude statements. There was no evidence of difference in overall attitude score between groups (see Table 2).

#### 3. Perceived confidence in appraising a published paper

There was no evidence of a statistically significant difference between groups in total confidence score (see Table 2).

#### 4. Ability to appraise a systematic review

There was some evidence of the ability of participants in the CAS training group to appraise 'results' of the systematic review article [ITT mean difference: 1.2 (95% CI: 0.01 to 2.4)]. However, the difference was not significant when assessed using explanatory analysis. No difference between groups was observed in the ability to appraise 'methodology' or 'relevance/generalisability' of evidence (see Table 2).

#### 5. Reading and evidence seeking behaviour

A comparison of various aspects of evidence seeking behaviour is detailed in Tables 3 and 4. The participants in the CAS training group self reported to: (1) read more articles, both for keeping up-to-date and for solving healthcare problems; (2) spend less time reading professional literature for keeping up-to-date, but spend more time reading professional literature for solving healthcare problems; (3) read 'thoroughly' a higher proportion of articles; and (4) use of the Cochrane library more frequently and, (5) read research reports, textbooks and other resources less frequently for solving healthcare problems. However, with the exception of (4), none of these differences were statistically significant in comparison to control

**Table 3:** CAS training and control groups reported number of articles read, and number of hours spent reading.

	**CAS training Mean (SD)**	**Control Mean (SD)**	**Intention to treat Mean difference+ (95% CI)**	**Explanatory analysis Mean difference+ (95% CI)**
**No. articles looked at or read thoroughly each week for keeping up-to-date**	5.7 (6.4)	5.1 (4.3)	0.9 (-0.6 to 1.2)	0.5 (-0.7 to 1.3)
**No. hours spent reading professional literature each week for keeping up-to-date**	2.2 (1.9)	2.5 (3.9)	0.9 (-0.6 to 1.2)	0.9 (-0.6 to 1.3)
**No. articles looked at or read thoroughly each week to solve a health care problem**	1.1 (0.8)	0.9 (0.8)	1.5 (-0.8 to 2.7)	1.4 (-0.8 to 2.7)
**No. hours spent reading professional literature to solve a health care problem**	0.9 (0.7)	0.9 (0.6)	-0.02 (-0.4 to 0.3)	-0.1 (-0.5 to 0.2)
**Proportion of articles read thoroughly**	21.9 (23.6)	19.2 (19.9)	1.3 (-0.8 to 2.0)	2.6 (-0.7 to 1.8)
**Proportion of articles skim read**	37.0 (20.8)	42.3 (24.9)	-5.7 (-15.4 to 4.1)	-8.2 (-18.1 to 1.6)
**Proportion of articles for which only abstracts read**	49.7 (23.4)	40.8 (26.7)	7.9 (-3.3 to 19.1)	12.0 (1.0 to 23.0)*

+ Adjusted for sex, age, attendance at previous educational activity, access to medical library, prior experience of searching literature, formal education in research methods and/or epidemiology and or statistics, prior involvement in research

* Statistically significant at *P *≤ 0.05

**Table 4:** CAS training and control groups use of the resources for solving a health care problem

	**CAS training Median (LQ, UQ)**	**Control Median (LQ, UQ)**	**Median Difference (p-value) †**
**Review articles**	2.0 (1.0, 3.0)	2.0 (1.25, 2.0)	0 (0.66)
**Research reports**	1.0 (1.0, 2.0)	2.0 (1.0, 2.0)	1.0 (0.97)
**Secondary journals**	2.0 (1.0, 3.0)	2.0 (1.0, 2.0)	0 (0.22)
**Textbooks**	2.00 (2.0, 3.0)	3.0 (1.0, 3.0)	1.0 (0.77)
**Worldwide Web**	1.0 (0, 2.0)	1.0 (0, 2.0)	0 (0.98)
**Guidelines**	2.0 (2.0, 3.0)	2.0 (2.0, 3.0)	0 (0.64)
**Cochrane Library**	1.0 (0, 2.0)	0 (0, 1.0)	1.0 (0.05)
**Colleagues**	3.0 (2.75, 3.0)	3.0 (2.0, 3.0)	0 (0.55)
**Other resources**	2.0 (0, 3.0)	3.0 (1.5, 3.75)	1.0 (0.41)

† Mann Whitney test; Likert Scale: '0': 'never'; '1': 'rarely'; '2': 'occasionally'; '3': 'often' & '4': 'very often'; UQ: upper quartile; LQ: lower quartile

### Costs

The mean cost to the NHS of conducting the CAS workshops was £250 per person (see Table 5). The majority of this cost (approximately £140) resulted from salary costs associated with the time of the participants attending the workshop. The remaining costs of the workshops were associated with the administration (approximately £25 per person), venue hire (approximately £42 per person), and tutors' time and travel (approximately £49 per person). There was some variation in the cost (from approximately £240 – £340 per person) across the 7 workshops, due to the attendance level, i.e. workshops with the most participants tended to have the lower cost.

**Table 5:** Summary of costs of CAS training

**Workshop**	**I**	**II**	**III**	**IV**	**V**	**VI**	**VII**	**Total**	**Total per Head**
**No. Attendees**	**16**	**19**	**9**	**14**	**18**	**5**	**7**	**88**	
Administration*	£322	£329	£313	£318	£316	£295	£300	£2,193	£25
**Venue**	£1028	£670	£285	£916	£475	£215	£74	£3,663	£42
**Participants' costs†**	£2,074	£2,825	£1,567	£1,878	£2,179	£793	£992	£12,310	£140
**Tutors' costs**	£709	£719	£890	£712	£555	£636	£73	£4,294	£49
**Total**	£4,132	£4,542	£3,057	£3,824	£3,525	£1,940	£1,439	£22,460.	£255
**Total per head**	£258	£239	£340	£273	£196	£388	£206	£255	

* Costs of initial invitations, copyright permission for use of paper, invitations to participants, pre workshop & post workshop pack production, postage costs, preparation for workshop, and travel bookings.

† Costs included participants' time at workshop with the exception of GPs, in these cases the cost of locum cover was applied.

## Discussion

The results of this prospective randomized controlled trial demonstrates that a half-day CAS workshop can elicit small improvements in healthcare professionals' knowledge of the principles and theory of evidence-based practice and some improvement in aspects of their critical appraisal skills ability. Nevertheless, we found little evidence of any improvement, as a result of CAS training, in the other study outcomes, i.e. participants' attitude towards evidence or their evidence seeking behaviour. Taking into account the set up costs and of time and locum expenses of participants, the mean cost of conducting these CAS training workshops was about £250 per person. The lack of substantive improvements in knowledge, skills and attitudes outcome observed in this trial are consistent with previous studies of CAS training [6-9].

### Potential limitations of this study

The number of participants recruited was less than that intended, not all participants provided outcomes and the trial was about 20 percent under the desired power. Nevertheless this study remains the largest randomized controlled trial to date and some statistically significant differences were observed.

The educational context in which this randomized trial was undertaken imposed certain constraints on its conduction and execution. As a result, poor recruitment, loss to follow up and poor uptake of the CAS training experienced by this trial may have threatened both its internal validity and generalisability. However, efforts were made in the analysis of the findings of this trial to overcome these limitations. The return of outcomes in this trial could not be mandatory. Despite considerable efforts by the project team (reminders and personal telephone calls from the trial principle investigator to participants), we failed to obtain a substantial proportion of outcomes in the trial participants – 60% and 85% of the knowledge, attitude and behaviour outcomes were obtained for CAS training and control groups respectively, and even less for the critique of the published systematic review. It is plausible that respondents may have differed in some way to non-respondents, such as in their level of motivation, and may therefore responded more positively to this educational intervention. However, this was not supported by the poor outcome response rate. Moreover there was no evidence of a difference in the baseline characteristics of participants who returned their outcomes, and those who did not. A differential response rate across the two study groups possibly reflects a greater reluctance in those individuals who had undertaken the educational intervention to return their outcomes (i.e. 'more to lose') compared to those in the control group. If true, the direction, in terms of over- or underestimating the impact of the intervention, is uncertain. An interview-administered assessment, rather than a mail based one, may have enhanced outcome response rate.

Of the 73 participants allocated to receive CAS training only 52 (71%) actually attended. The reasons for this were unclear, and were not formally addressed within this study. In addition to conventional intention-to-treat analyses, secondary explanatory analyses, i.e. based upon the participants who actually did attend the workshop, were undertaken. That there were no differences between groups for most outcomes, irrespective of whether an intention-to-treat or explanatory analysis, was used (see Tables 2 and 3) suggests that the poor intervention uptake was not important source of bias.

### Implications of findings

With the drive to evidence-based practice in recent years, considerable efforts have been made in providing CAS training as part of healthcare professionals' undergraduate and postgraduate activities in many countries. The findings of this study, the largest randomized controlled trial to date, provide only limited support for such training. However, it is important to put this finding in the appropriate educational context. The half-day CASP workshop evaluated in this trial has been widely disseminated and its duration and format is consistent with many previous CAS interventions [9]. Nevertheless it is probably unrealistic to expect that the half-day workshop evaluated in this trial would in itself result in changes in professional behaviour. This is supported by a large body of evidence and theory on changing professional practice [17]. Therefore it is important to see, and assess, CAS training, not in isolation, but as one part of education approach towards evidence-based practice or as a part of the undergraduate and postgraduate curriculum. It is also important to reassess the objective of CAS training. With increasing availability of carefully appraised evidence such as secondary journals (e.g. *Evidence Based Medicine*) and on-line critically appraised topics ('CATs'), the most important role of CAS training may be simply be to sensitise participants to the availability of high quality evidence. Further debate is therefore needed about refocusing critical appraisals skills training towards finding such evidence and the role of healthcare librarians and the new initiatives such as the National Electronic Library for Health. A number of commentators have criticised previous evaluations of CAS training for not using experimental designs [6-9]. However, the experience of this study has demonstrated some of the difficulties in implementing an evaluation of 'real life' educational intervention using such an experimental design. The difficulty of employing randomized controlled trials in the evaluation of educational interventions has been highlighted by others [18]. Future evaluations of CAS and other educational interventions aimed at promoting evidence-based practice need to take into account both these perspectives.

## Conclusions

This prospective randomized controlled found small improvements in self-selected healthcare professionals' knowledge and understanding of the medical literature and appraisal skills with critical appraisal skills training. No improvement was observed in attitudes towards the use evidence and evidence-seeking behaviour. The findings of this study challenge the policy of funding in isolation 'one-off' educational interventions aimed at enhancing the evidence-based practice of health care professionals. Future evaluations of evidence-based practice interventions need to take in account both this trials' negative findings and methodological difficulties.

## List of abbreviations

CAS – critical appraisal skills

CASP – Critical Appraisals Skills Programme

95% CI – 95 percent confidence interval

ITT – intention to treat

NHS – National Health Service

R&D – research and development

## Competing interests

The author(s) declare that they have no competing interests.

## Author's contributions

RT, BR and PE conceived, designed and secured funding for the trial. RST drafted the paper. RJT collected the study outcomes and undertook the data analysis. RST is a guarantor for the study.

## Funding

NHS R&D Executive: Evaluating methods to practice the implementation of R&D [project no. IMP 12-9]

## Appendix 1. Objectives, syllabus, and delivery methods of critical appraisal skills workshop for health care decision makers

### Workshop objectives *(taken from workshop materials)*

• To critically appraised a published review article.

• To understand the terms systematic review and meta-analysis.

• To be able to explain why critical appraisal skills are important for provision of health care.

• To have greater confidence in your ability to make sense of the research evidence.

### Workshop format

3 hours attendance (also advised to undertake at least 1 hour preparation reading the article to be appraised in the workshop and address a written 'clinical scenario')

• Introductory talk: overview of the importance of evidence based health care practice, the theoretical basis of the appraisal of a systematic review, and orientation to the *JAMA *appraisal guideline (~60 mins).

• Small group work: appraisal of a published systematic review (~60 mins).

• Plenary session: feedback from the small group, general discussion of the relevance of the appraisal to clinical scenario and ballot of opinions on the clinical scenario. (~60 mins)

All workshops were run by 3 to 4 individuals each of whom had a formal training in health services research methods and were experienced in delivering CASP workshops.

### Workshop materials

One to two weeks prior to the workshop, a pre-workshop pack was sent to participants.

• Workshop objectives.

• Orientation guide.

• Clinical scenario and questions

• Systematic review paper.

• Glossary.

One to two weeks post workshop, a post workshop pack was sent to participants:

• Introductory talk slides.

• Systematic review checklist.

• *JAMA *guidelines for systematic review [15].

### Educational rationale

The workshop is based on the Critical Appraisal Skills Programme (CASP) developed by Oxford Regional Health Authority and developed from the educational methods of McMaster University in Canada [5]. The 'McMaster model' key features include, self-directed learning, small group teaching methods and the importance of grounding education within the clinical decision making process.

## Pre-publication history

The pre-publication history for this paper can be accessed here:



## Supplementary Material

Additional File 1 MS-Word format, contains study outcome questionnaire Click here for file
