# Supplementary material for: Critical appraisal skills training for health care professionals: a randomized controlled trial [ISRCTN46272378]
Source: BMC Med Educ. 2004 Dec 7;4:30. doi: 10.1186/1472-6920-4-30 (PMC539272; doi:10.1186/1472-6920-4-30)
Supplement: Additional File 1 — MS-Word format, contains study outcome questionnaire [file 1472-6920-4-30-S1.doc]

**Appendix 1. Objectives, syllabus, and delivery methods of critical appraisal skills workshop for health care decision makers**

**Workshop objectives** *(taken from workshop materials)*

- To critically appraised a published review article.
- To understand the terms systematic review and meta-analysis.
- To be able to explain why critical appraisal skills are important for provision of health care.
- To have greater confidence in your ability to make sense of the research evidence.

# Workshop format

*3 hours attendance (also advised to undertake at least 1hour preparation reading the article to be appraised in the workshop and address a written ‘clinical scenario’)*

- Introductory talk: overview of the importance of evidence based health care practice, the theoretical basis of the appraisal of a systematic review, and orientation to the *JAMA* appraisal guideline (~60 mins).
- Small group work: appraisal of a published systematic review (~60mins).
- Plenary session: feedback from the small group, general discussion of the relevance of the appraisal to clinical scenario and ballot of opinions on the clinical scenario. (~60 mins)

All workshops were run by 3 to 4 individuals each of whom had a formal training in health services research methods and were experienced in delivering CASP workshops.

# Workshop materials

One to two weeks prior to the workshop, a pre-workshop pack was sent to participants.

- Workshop objectives.
- Orientation guide.
- Clinical scenario and questions
- Systematic review paper.
- Glossary.

One to two weeks post workshop, a post workshop pack was sent to participants:

- Introductory talk slides.
- Systematic review checklist.
- *JAMA* guidelines for systematic review ^15^.

# Educational rationale

The workshop is based on the Critical Appraisal Skills Programme (CASP) developed by Oxford Regional Health Authority and developed from the educational methods of McMaster University in Canada [5]. The ‘McMaster model’ key features include, self-directed learning, small group teaching methods and the importance of grounding education within the clinical decision making process.

**Appendix 2. Outcome questionnaire**

**CRITICAL APPRAISAL SKILLS PROGRAMME**

**WORKSHOP EVALUATION QUESTIONNAIRE**

Thank you for taking the time to complete this questionnaire. Whether you have attended a CASP workshop or not, your answers will help us to evaluate the workshops.

**There are four sections, each with different instructions.** **As the scoring system in each section is different** **please read the instructions carefully.** **The shaded boxes are for office use only.**

**Your answers will only be used to evaluate the workshops, and all responses will be treated confidentially.**

| 1. **YOUR BACKGROUND** | | | **Office use only** | |
| --- | --- | --- | --- | --- |
|  |  |  |  | |
|  |  |  |  | |
| **Please tick the appropriate box:** | **YES** | **NO** |  | |
| 1. Have you ever attended a Critical Appraisal Skills (CASP) workshop? If yes, how long ago? ……………weeks/months/years ^*^delete as appropriate | **🞏** | **🞏** |  |  |
| 1. Have you attended any other professionally approved (e.g. PGEA) educational activity in the last six months? | **🞏** | **🞏** |  | |
| 1. Do you have access to a staffed medical/health care library? If so, how long does it take you to get there?………………….minutes. | **🞏** | **🞏** |  |  |
| 1. Do you have access to literature via the Internet and/or Telnet? | **🞏** | **🞏** |  | |
| 1. Have you searched the literature for any form of published evidence in the last six months? | **🞏** | **🞏** |  | |
| 1. Have you received any formal education or training (eg. degree course, seminar, workshop) in any of the following?   a) Research methods  b) Epidemiology  c) Statistics | **🞏**  **🞏**  **🞏** | **🞏**  **🞏**  **🞏** |  | |
| 7. Have you personally been involved in conducting any kind of research? | **🞏** | **🞏** |  | |

**B. USING EVIDENCE**

**INSTRUCTIONS:**

**Please answer the following questions, which refer to your use of evidence in decision-making**

|  | 1. For browsing/keeping up to date | 1. To solve a health care problem | **Office use only** | |
| --- | --- | --- | --- | --- |
| 1. On average, how many journal articles do you look at or read thoroughly each week? | …………………………. | ……………………… |  |  |
| 1. On average, how many hours per week you spend reading your professional literature? | …………………………. | ……………………… |  |  |

|  |  |  | | **Office use only** |
| --- | --- | --- | --- | --- |
| 1. Of the articles that you look at, | a) Read thoroughly? | | .…..……% |  |
| what proportion do you: | b) Skim? | | …………% |  |
|  | c) Read the abstract only? | | …….…..% |  |

| 4. What types of resource do you use to keep up to date? | | | | | | |  |
| --- | --- | --- | --- | --- | --- | --- | --- |
|  | **Please tick the appropriate box for each option.** | **Never** | **rarely** | **occasionally** | **often** | **very often** | **Office use only** |
|  | 1. Journals: review articles |  |  |  |  |  |  |
|  | 1. Journals: original research reports |  |  |  |  |  |  |
|  | 1. Secondary journals eg. *Evidence Based Medicine/Nursing, Bandolier* |  |  |  |  |  |  |
|  | 1. Textbooks |  |  |  |  |  |  |
|  | 1. Internet resources/computer databases or similar |  |  |  |  |  |  |
|  | 1. Clinical guidelines |  |  |  |  |  |  |
|  | 1. The Cochrane Library |  |  |  |  |  |  |
|  | 1. Colleagues |  |  |  |  |  |  |

| 5. What types of resource do you use to solve a specific health care problem? | | | | | | |  |
| --- | --- | --- | --- | --- | --- | --- | --- |
|  | **Please tick the appropriate box for each option.** | **never** | **rarely** | **occasionally** | **often** | **very often** | **Office use only** |
|  | 1. Journals: review articles |  |  |  |  |  |  |
|  | 1. Journals: original research reports |  |  |  |  |  |  |
|  | 1. Secondary journals eg. *Evidence Based Medicine/Nursing, Bandolier* |  |  |  |  |  |  |
|  | 1. Textbooks |  |  |  |  |  |  |
|  | 1. Internet resources/computer databases or similar |  |  |  |  |  |  |
|  | 1. Clinical guidelines |  |  |  |  |  |  |
|  | 1. The Cochrane Library |  |  |  |  |  |  |
|  | 1. Colleagues |  |  |  |  |  |  |
|  | 1. Other resource (please specify) …………………….................... |  |  |  |  |  |  |

6. How confident do you think that you are at assessing each of these aspects of a published paper?

| **Please tick the box that best represents your level of confidence.** | **Very confident** | **Quite confident** | **Moderately confident** | **Not very confident** | **Not at all confident** | **Don’t know** | **Office use only** |
| --- | --- | --- | --- | --- | --- | --- | --- |
| a) Assessing study design |  |  |  |  |  |  |  |
| b) Evaluating bias |  |  |  |  |  |  |  |
| c) Evaluating the adequacy of sample size |  |  |  |  |  |  |  |
| d) Assessing generalisability |  |  |  |  |  |  |  |
| e) Evaluating statistical tests/principles |  |  |  |  |  |  |  |
| f) Assessing the general worth of an article. |  |  |  |  |  |  |  |

**C. Your Views**

**INSTRUCTIONS:**

Below are 7 statements about critical appraisal and using evidence.

**Please read each statement carefully and then tick the box that reflects your views most closely.**

|  | **I strongly agree** | **I**  **agree** | **I don’t agree or disagree** | **I**  **disagree** | **I strongly disagree** | **I don’t know** | **Office use only** |
| --- | --- | --- | --- | --- | --- | --- | --- |
| 1. Original research is confusing. |  |  |  |  |  |  |  |
| 1. Study design is important in article selection. |  |  |  |  |  |  |  |
| 1. Evidence-based decision-making is “health care by numbers”. |  |  |  |  |  |  |  |
| 1. NHS contracts for health care professionals should include time taken away from patient care for reading and appraising the literature. |  |  |  |  |  |  |  |
| 1. I am confident that I can assess research evidence. |  |  |  |  |  |  |  |
| 1. Systematic reviews play a key role in informing evidence-based decision-making. |  |  |  |  |  |  |  |
| 1. The NHS should have its own programme of research about clinical effectiveness. |  |  |  |  |  |  |  |

**D. Multiple Choice Questions**

**INSTRUCTIONS:** Below are 6 multiple choice questions about the application of critical appraisal skills.

**Please read each question and the accompanying statements carefully and then answer “true”, “false”, or “don’t know” for EACH statement (a, b, and c) by ticking the appropriate boxes.**

We are only interested in what you currently know. Please do not look up the answers, and please do not guess. Just tick the “don’t know” box if appropriate.

| 1. Meta-analysis of studies (pooling the data from several studies to obtain an ‘overall’ answer) in a systematic review of clinical effectiveness is often restricted to RCTs. By using RCTs, one can be more confident that: | **TRUE** | **FALSE** | **DON’T KNOW** | **Office use only** |
| --- | --- | --- | --- | --- |
| 1. the likely treatment outcomes of patients contributing to the meta-analysis are representative of the population for whom the treatment is intended. |  |  |  |  |
| 1. factors such as age and severity of disease, which may influence outcome, are balanced between patients allocated to the new and old treatments. |  |  |  |  |
| 1. any difference in treatment outcomes between patients receiving the new and old treatments can be attributed to the new treatment. |  |  |  |  |
| 2. An article reports on a new treatment for children who suffer from asthma, which may prevent them having any further asthma attacks in the following year. The article states that the number needed to treat (NNT) is 120. | **TRUE** | **FALSE** | **DON’T KNOW** | **Office use only** |
| 1. A study designed to evaluate the new treatment will need 120 patients to be sure of getting a significant result. |  |  |  |  |
| 1. On average, if 120 patients were treated then one of them will experience a further asthma attack within one year. |  |  |  |  |
| 1. On average, if 120 patients were treated then one child fewer will experience a further asthma attack within one year than if these patients were not treated. |  |  |  |  |
| 3. A systematic review suggests that a new treatment reduces the risk of death in the first year following a heart attack when compared to conventional management, with an odds ratio of 0.5. The 95% confidence interval is 0.2 to 1.2. | **TRUE** | **FALSE** | **DON’T KNOW** | **Office use only** |
| 1. There is a 95% certainty that the treatment is effective. |  |  |  |  |
| 1. There is a 95% certainty that the treatment is not effective. |  |  |  |  |
| 1. More research is desirable to narrow the confidence interval. |  |  |  |  |

| 4. A series of six trials have been included in a meta-analysis reporting on the benefits of acyclovir in the treatment of acute herpes zoster (shingles). | **TRUE** | **FALSE** | **DON’T KNOW** | **Office use only** |
| --- | --- | --- | --- | --- |
| 1. Before adopting a policy to use acyclovir for shingles it would be sensible to know in what settings (tertiary specialist centre, DGH, or community) the studies took place. |  |  |  |  |
| 1. If the settings were similar to your own, you are likely to be more confident about using the evidence. |  |  |  |  |
| 1. It is important to consider whether any of your own patients with shingles would have been excluded from the studies. |  |  |  |  |
| 5. When combining the results of many studies in a systematic review, there are many issues to consider. | **TRUE** | **FALSE** | **DON’T KNOW** | **Office use only** |
| 1. Studies that have sample sizes that are too small to detect a clinically important benefit should be included. |  |  |  |  |
| 1. If a small number of studies are found to yield answers which are dramatically different from the majority they should be excluded. |  |  |  |  |
| 1. Publication bias is a tendency for doctors to prefer one treatment over another because there has been publicity on the issue. |  |  |  |  |
| 6. A systematic review investigating the effectiveness of a new antibiotic for treatment of otitis media reports on eight randomised trials. Each trial evaluated the therapy using the same outcome measure. Four of the studies report a statistically significant benefit of using the new treatment, while the remaining four report that there was no statistically significant benefit. | **TRUE** | **FALSE** | **DON’T KNOW** | **Office use only** |
| 1. We can conclude from this that the eight studies are incompatible. |  |  |  |  |
| 1. One plausible reason for the findings is that the antibiotic is beneficial but the non-significant studies had inadequate numbers of patients. |  |  |  |  |
| 1. One plausible reason for the findings is that the antibiotic is not beneficial but the studies claiming a benefit contained biases. |  |  |  |  |

**THANK YOU FOR COMPLETING THE QUESTIONNAIRE**
